# Evaluating a Web-Based Application to Facilitate Family-School-Health Care Collaboration for Children With Neurodevelopmental Disorders in Inclusive Settings: Protocol for a Nonrandomized Trial

**DOI:** 10.2196/63378

**Published:** 2025-04-17

**Authors:** Eric Meyer, Hélène Sauzéon, Isabeau Saint-Supery, Cecile Mazon

**Affiliations:** 1 GRHAPES (Research group on disability, accessibility and educational practices, UR 7287) Institut national supérieur de formation et de recherche pour l'éducation inclusive (INSEI) Suresnes France; 2 Flowers team-project Inria Research Center of the University of Bordeaux Talence Cedex France; 3 ACTIVE team Inserm-University of Bordeaux Bordeaux Population Health (U1219) Bordeaux France

**Keywords:** neurodevelopmental disorders, coeducation, whole-school approach, family-professional partnership, web application, inclusive education, family-school-health care

## Abstract

**Background:**

An individual education plan (IEP) is a key element in the support of the schooling of children with special educational needs or disabilities. The IEP process requires effective communication and strong partnership between families, school staff, and health care practitioners. However, these stakeholders often report their collaboration as limited and difficult to maintain, leading to difficulties in implementing and monitoring the child’s IEP.

**Objective:**

This paper aims to describe the study protocol used to evaluate a technological tool (CoEd application) aiming at fostering communication and collaboration between family, school, and health care in the context of inclusive education.

**Methods:**

This protocol describes a longitudinal, nonrandomized controlled trial, with baseline, 3 month, and 6-month follow-up assessments. The intervention consisted of using the web-based CoEd application for 3 months to 6 months. This application is composed of a child’s file in which stakeholders of the support team can share information about the child’s profile, skills, aids and adaptations, and daily events. The control group is asked to function as usual to support the child in inclusive settings. To be eligible, a support team must be composed of at least two stakeholders, including at least one of the parents. Additionally, the pupil had to be aged between 10 years and 16 years, enrolled in secondary school, be taught in mainstream settings, and have an established or ongoing diagnosis of autism spectrum disorder, attention-deficit/hyperactivity disorder, or intellectual disability (IQ<70). Primary outcome measures cover stakeholders’ relationships, self-efficacy, and attitudes toward inclusive education, while secondary outcome measures are related to stakeholders’ burden and quality of life, as well as children’s school well-being and quality of life. We plan to analyze data using ANCOVA to investigate pre-post and group effects, with a technological skills questionnaire as the covariate.

**Results:**

After screening for eligibility, 157 participants were recruited in 37 support teams, composed of at least one parent and one professional (school, health care). In September 2023, after the baseline assessment, the remaining 127 participants were allocated to the CoEd intervention (13 teams; n=82) or control condition (11 teams; n=45).

**Conclusions:**

We expect that the CoEd application will improve the quality of interpersonal relationships in children’s IEP teams (research question [RQ]1), will show benefits for the child (RQ2), and improve the well-being of the child and the stakeholders (RQ3). Thanks to the participatory design, we also expect that the CoEd application will elicit a good user experience (RQ4). The results from this study could have several implications for educational technology research, as it is the first to investigate the impacts of a technological tool on co-educational processes.

**International Registered Report Identifier (IRRID):**

DERR1-10.2196/63378

## Introduction

### Background

There is a growing body of literature on the benefits of using educational technologies to support children with special educational needs or disabilities (SEND) and, notably, those due to neurodevelopmental disorders (NDD; eg, [1-4]). Most of these technologies target students as their primary users, forgetting the social environment that has to work together to support the children in their educational project. This study aims to conduct a field study to assess the effectiveness of an interactive web application (namely, the CoEd application) for fostering collaboration between family, school, and health care practitioners.

In several countries, a key element in the support of the child’s schooling is the individual education plan (IEP), which defines their needs and gathers information on aids and adaptations that can be implemented to help them attain their full potential. IEP structures differ by country, but the core process emphasizes a robust partnership among families, school staff, and health care professionals, with regular meetings and a deep understanding of the child's profile and progress [5,6]. Effective communication between teachers and parents is universally recognized as a cornerstone of a child’s education [6,7]. The ability of parents to share pertinent information about their child’s disabilities, strengths, and weaknesses significantly enhances educators’ awareness and enables them to tailor their pedagogical approaches [8]. Furthermore, parental involvement in the educational process has far-reaching implications, encompassing the holistic development and well-being of children with SEND. It serves as the bedrock for fostering a culture of productive family-school collaboration and cultivating a supportive school environment [9,10]. For students grappling with complex disorders such as autism spectrum disorder (ASD), the synergy of collaboration between parents and educators paves the way for improved academic performance, smoother integration, and enhanced social adaptation [11]. In a broader context, collaboration between parents and educators emerges as a catalyst for positive outcomes, benefiting students across the spectrum of educational needs [12]. Despite all of this, studies on the IEP process consistently underscore the pronounced discord between stakeholders’ aspirations for effective teamwork and the actual communication and collaboration practices in place [12-17]. This paradox is particularly evident in the experiences of parents, teachers, and educators who frequently encounter obstacles ranging from resource constraints to training deficits, communication impediments, intercoordination challenges, and intricate administrative processes [12,18-22]. These challenges are not unique to inclusive education of children with NDD and extend to other areas such as patient care [23,24].

Within the continuum of collaborative practices in inclusive education, 3 progressive levels can be delineated: cooperation, coordination, and collaboration [25]. As elucidated by Larivée et al [26], elevation in the school-family-community collaboration level correlates with mutual recognition of expertise, bidirectional communication, shared responsibilities, and the spirit of reciprocity.

Several theoretical models of collaborative practices within the framework of IEPs have been proposed. These models may pivot around the child, exemplified by the “Whole School, Whole Community, Whole Child” model [27,28], or emphasize the dynamics of partnerships between stakeholders, as exemplified by the Sunshine model [29]. Other models are grounded in the objectives of IEPs, as demonstrated by polycentric approaches based on the child’s life project [30]. Additionally, some models embrace a multidimensional perspective typified by the Holistic School Care Coordination System Model [31,32].

The proliferation of these models underscores the pivotal role of educational systems in facilitating interactions between educators and families. This entails the provision of guidelines and recommendations for effective collaboration, alongside the allocation of critical resources encompassing time, physical infrastructure, and financial support [19,20]. Among the envisaged solutions, harnessing digital technologies emerges as a promising avenue to support continuous communication for efficient stakeholder collaboration [33,34].

### Prior Work

Maintaining communication and collaboration within the extended team for IEPs is challenging due to various communication channels like emails and phone calls. Despite digital advancements, tools often prioritize one-to-one communication over team-based sharing. Additionally, the lack of trackability in one-to-one exchanges hampers information flow within the team. To the best of our knowledge, very few digital tools are designed to help stakeholders with team-based information sharing, communication, and collaboration in the follow-up of the IEP and of the child’s schooling and development [35]. Many proposed devices in this field lack maturity, either due to an inadequate design that does not consider user needs, resulting in usability issues, or because participatory design methods were used without subsequent effectiveness studies conducted to standard methodological levels for producing evidence (for a review, see [35]). An example of that is illustrated by the CoEd application, which provides a web application with functionalities aiming at fostering information sharing, communication, and collaboration in IEP and with the support team [36]. Through participatory methods, the CoEd application was designed based on stakeholders’ needs and iterative design steps for eliciting good user experience, usability, and utility (see [36] for the design process).

### Aims

We propose to conduct a field study aiming to evaluate the impact of using the CoEd web application on 3 outcomes. First, we hypothesized that the CoEd application will improve communication and relationships between stakeholders (family, school, and health care practitioners), as well as their attitudes toward school inclusion, and increase each stakeholder’s perceived self-efficacy (research question [RQ]1). Second, we hypothesized a beneficial effect of using CoEd on the child in terms of behavioral or academic functioning (RQ2). Third, we expect that using CoEd results in a better quality of life for both the stakeholders and the children (RQ3). Finally, as it is crucial when evaluating technology to verify that it is both effective and usable [37], we added measures related to the user experience of participants. Thanks to the participatory design, we expected that the CoEd application will elicit a good user experience (RQ4).

The paper follows the SPIRIT (Standard Protocol Items: Recommendations for Interventional Trials) guidelines for reporting study protocols [38]. The SPIRIT checklist for this paper is provided in Multimedia Appendix 1.

## Methods

### Ethical Considerations

The ethics committee (Comité Opérationnel d'Évaluation des Risques Légaux et Éthiques [COERLE]) of the French National Institute of Informatics and Mathematics (Inria) approved the study protocol (application number 2022-08).

As the study protocol implies the management of health-related data, the CoEd app is hosted and managed on a secured server at Inria as if CoEd’s data are medical data, in respect of the General Data Protection Regulation (GDPR). Additionally, the study protocol and data management processes were approved by the National Commission of Informatics and Liberty and by the Inria Security Homologation committee (application number 13953). All participants were informed about the study and gave written informed consent.

### Study Overview

We designed a longitudinal, nonrandomized controlled trial to evaluate the impact of the CoEd application developed by our team [36]. We enrolled teams monitoring the school or social inclusion of secondary school pupils with NDD (between 10 years and 16 years old). These teams were recruited throughout France via email communication with schools, school districts, special education advisors, academic inspectors specializing in Special Education, and associations supporting families of children with NDD. Field partners of the project, namely the Nouvelle-Aquitaine academy, Autism Resources Center, and Association pour la Réadaptation et l'Intégration, were also engaged in the recruitment process and encouraged participation in their networks. We also put out calls for participants on digital social networks. Parents, teachers, and health care professionals were asked to complete a questionnaire to check the inclusion criteria. When the participant and child were eligible, all the members of the follow-up team were contacted, and the study was presented to them. Once participation was confirmed, individual participants received a consent form. They were given a week to return the completed form, with researchers available to address any questions during this period.

**Figure 1:**
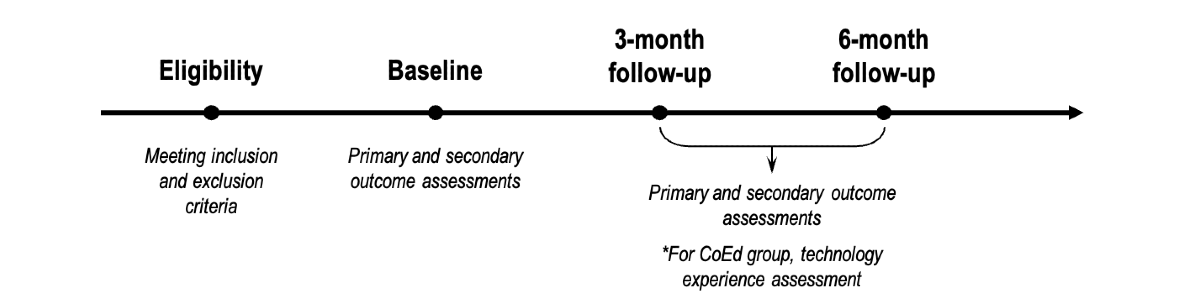
Timeline of the field study according to the group conditions and the number of school terms (3 months for each term) for study participation.

Whether they were in the control or experimental condition, they had to complete a baseline assessment and a 3-month follow-up assessment, and if they were willing to continue participating, they would complete a 6-month follow-up assessment (Figure 1).

### Eligibility Criteria

To participate, a support team had to consist of at least two stakeholders, including at least one of the parents. Additionally, the pupil had to be aged between 10 years and 16 years, enrolled in secondary school, be taught in mainstream settings, and have a diagnosis of ASD, attention-deficit/hyperactivity disorder, or intellectual disability (IQ<70). The pupil needed either an established diagnosis or to be undergoing a multidisciplinary intervention for diagnosis.

### Allocation and Blinding

In this quasi-experimental trial, the group allocation was not randomized. Group allocation was carried out as participants were recruited according to eligibility criteria, taking care to balance groups in terms of team size. A nonrandomized procedure was chosen because we recruited participants as volunteers arose who had a complete team. As the control group did not use the CoEd application and functioned as usual whereas the experimental group was asked to use the CoEd application and needed secure access to the web-based application, it was not possible to conduct a double-blind study.

### Outcome Measures

#### Selection of Outcome Measures

Outcome measures were chosen from the researchers’ hypotheses and participatory decision-making. In 2 focus groups, parents, teachers, and health care professionals discussed the CoEd application’s potential impact and evaluation criteria (Multimedia Appendix 2). Participants reviewed preselected indicators and suggested new measures, especially concerning quality of life and caregiver burden, for the field study. As a result, the primary outcomes include 3 main dimensions (ie, stakeholders’ relationships, perceived individual efficacy, and attitudes toward school inclusion). To this end, parents, teachers, and health care professionals completed a series of common or specific questionnaires (Table 1). The secondary outcomes include measures for stakeholders (burden and quality of life) and for the children (quality of life, school well-being, and school inclusion perception; Table 1). Additionally, a measurement of technology experience that covers technology skills and CoEd experience was included for the intervention group (Table 1). Although some questionnaires had a validated French version, others did not. In the latter case, as we are French native speakers, we translated them in collaboration with native English speakers. The scales used are presented in Table 1.

**Table 1:** Description of the assessment batteries to address the targeted (primary and secondary) outcomes and technology experience of participants.

Outcomes, dimension, and subdimension				Questionnaire		Respondent						
						Child		Parent		Teacher		Professionals
**Primary outcomes (stakeholders)**												
	**Relationship**											
		Parent-teacher	Parent-Teacher Relationship Scale II^a^		—^b^		X		X		—	
		Therapeutic or interprofessional	Helping Alliance Questionnaire		—		X		X		X	
	**Perceived educational efficacy**											
		Self-efficacy	Basic Psychological Need: Satisfaction and Frustration scale		—		X		X		X	
		Achievement of educational goals	Goal Attainment Scale		—		X		X		X	
	**Attitudes toward school inclusion**											
		Teachers	Multidimensional Attitudes Towards Inclusive Education Scale		—		—		X		—	
		Parents	Attitudes Toward Inclusion/Mainstream^a^		—		X		—		—	
**Secondary outcomes (stakeholders)**												
	**Burden**											
		Family burden	Caregiver Strain Questionnaire		—		X		—		—	
		Adult burden	Zarit Inventory Short Form		—		—		X		X	
		Burnout	Maslach Burn-out Inventory^a^		—		X		X		X	
	**Quality of life**											
		Adult	WHOQOL-Bref		—		X		X		X	
		Family	Beach Center Family Quality of Life (FQOL)		—		X		—		—	
**Secondary outcomes (children)**												
	**School life**											
		Child’s quality of life	Pediatric Quality of Life Inventory (PedsQL)		X		X		—		—	
		School well-being	Multidimensional Students' Life Satisfaction Scale (MSLSS)		X		—		—		—	
		School inclusion	Individual school perception		X		—		—		—	
**Technology experience^c^**												
	**Technology skills**											
		Computer skills	Computer Usage Questionnaire		—		X		X		X	
	**CoEd** **application experience**											
		Subjective user experience	User Experience Questionnaire, short version and Technology-based Experience of Need Satisfaction (TENS)^a^		—		X		X		X	
		Objective user experience	The uses and usages of the CoEd application are quantified with the following indicators across periods of 3 months or 6 months: (1) number of times the application is opened and (2) number of exchanged posts		—		X		X		X	

^a^Translated in French by the authors.

^b^Not applicable.

^c^Intervention group only.

#### Primary Outcomes

##### Perception of Stakeholders’ Relationships

Parents and teachers completed the Parent-Teacher Relationship Scale [39], which consists of a 24-item questionnaire measured on a 5-point Likert scale (1=strongly disagree to 5=strongly agree) assessing the 2 dimensions joining and communication-to-other, including their feeling of affiliation and support; the dependability and availability of both parties; shared expectations; and beliefs about the child and each other, their communication, and their sharing of information and emotions. To assess the relationship with medical-social professionals, the Helping Alliance Questionnaire [40] was completed by parents, teachers, and health care practitioners. The Helping Alliance Questionnaire initially evaluated the therapeutic alliance through the 2 dimensions perceived helpfulness and collaboration, but it was adapted to the purpose of the study. Two versions were used: a 13-item parent form and an 11-item professional form, both using a 6-point Likert scale (1=totally disagree to 5=totally agree).

##### Perceived Individual Efficacy

The feeling of self-determination was measured using the French version of the Basic Psychological Need Satisfaction and Frustration Scale [41], which assesses satisfaction and frustration with the 3 basic psychological needs: competence, affiliation, and autonomy. This 24-item scale using a 5-point Likert scale (1=Completely False to 5=Completely True) provides a proxy of general individual efficacy. A version of the Goal Attainment Scale [42] adapted by our team was proposed. Initially, parents and professionals were asked to independently specify 2 to 5 school, social, or behavioral goals for the young individual, along with their current level, desired level, difficulty, and importance. At the end of the experimentation, the goal was recalled, and the adults were asked to assess the level of goal attainment. This adapted version of the Goal Attainment Scale provides a probe of (individual) educational efficacy.

##### Attitudes Toward Inclusive Education

The parents’ attitudes toward inclusive education was measured using the Attitudes Toward Inclusion/Mainstream scale [43]. This questionnaire, consisting of 18 items that are rated on a 5-point Likert scale (1=strongly agree to 5=strongly disagree), assesses the overall attitude of parents toward the inclusion of students with disabilities in mainstream schools, including 4 dimensions (benefits factor, satisfaction with special education, teacher ability and inclusion support, child rights). The Multidimensional Attitudes Towards Inclusive Education Scale [44] was selected to evaluate teachers’ attitudes toward the inclusion of students with disabilities in mainstream schools. It consists of 18 items assessed using a 6-point Likert scale (1=strongly disagree to 6=strongly agree) and measuring 3 dimensions (cognitive, affective, behavioral).

#### Secondary Outcomes

##### Perception of Caregiver Burden

Parents and professionals completed the Zarit Burden Inventory Short Form [45,46], which consists of 12 items assessing the individual perception of their burden. Each item is scored between 1 point and 5 points (1=never to 5=almost always), and the total score is the sum of all item scores. The higher the score is, the higher the burden is, with the following cut-offs: no to mild burden (0-10), mild to moderate burden (10-20), and high burden (>20). For parents, the assessment was completed with the Caregiver Strain Questionnaire [47] to evaluate the familial burden through 21 items using a 5-point Likert scale (1=not at all to 5=very much) and 3 dimensions (objective burden, internalized subjective burden, and externalized subjective burden). The Caregiver Strain Questionnaire has been specifically validated with families of children with disabilities, including NDD or ASD. For professionals, the level of burnout was assessed using the French version of the Maslach Burnout Inventory [48], a 22-item scale using a 7-point Likert scale (1=never to 7=every day) that evaluates emotional exhaustion, depersonalization in relationships with others, and a sense of reduced personal accomplishment in one’s work.

##### Stakeholders’ Quality of Life

Parents and professionals completed the WHOQOL-BREF [49], a 26-item scale using a 5-point Likert scale (1=Very Low, Very Dissatisfied, Not at all, Very Difficult, Never to 5=Very High, Very Satisfied, Extremely, Very Easily, All the Time) recommended by the World Health Organization to assess individuals’ quality of life based on 4 dimensions (physical health, psychological health, social relationships, environment). The quality of life of the family was assessed using the Beach Center Family Quality of Life scale [50], adapted into French by Rivard et al [51]. This scale, consisting of 25 items using a 5-point Likert scale (1=Slightly Important, Very Dissatisfied to 5=Very Important, Very Satisfied), measures 5 dimensions of family quality of life: family interactions, parenting, emotional well-being, physical and material well-being, and disability-related support.

##### Child’s Quality of Life

The pupil’s quality of life was assessed using the Pediatric Quality of Life Inventory [52]. The Pediatric Quality of Life Inventory is composed of 23 items using a 5-point Likert scale (0=never to 4=almost always) assessing children’s physical, emotional, social, and school functioning through self-report (completed by children) and proxy report (completed by parents).

##### Child’s Perception of School Life

The student's sense of school inclusion was evaluated using the single-item inclusion scale by Aron et al [53], which measures 7 levels of the sense of inclusion and has been adapted to assess the sense of inclusion in both the school and peer group contexts. School satisfaction was evaluated using an 8-item subscale that uses a 7-point Likert scale (1=strongly disagree to 7=strongly agree) derived from the French adaptation of the Multidimensional Students’ Life Satisfaction Scale [54,55].

##### Measurement of Technology Experience (Experimental Group Only)

To assess the potential effect of prior technology skills on the use of CoEd, parents and professionals completed the Computer Usage Questionnaire [56] that consists of a self-reported measure of 18 questions asking for the frequency (5-point scale ranging from never to very often) of different computer activities and software usage.

To address the specific user experience with the CoEd application, the User Experience Questionnaire short form [57] was selected as the measurement. It uses a 7-point Likert scale (–3=fully agree with negative term to +3=fully agree with positive term) of subjective perceptions of users in terms of both the hedonic and pragmatic dimensions toward a given product and according to a benchmark for digital products. The Technology-based Experience of Need Satisfaction-Interface [58] is related to the self-determination theory framework for assessment and uses a 5-point Likert scale (1=Do Not Agree to 5=Strongly Agree) to assess basic psychological need (autonomy, competence, relatedness) satisfaction through the interaction with the interface or system. Satisfaction of these needs is arguably associated with sustained engagement with a technology.

Complementary to the subjective measures, direct measures of CoEd use and usage are collected through active interactions (log data) with this web application, in particular the number of times the app was opened and the number of exchanged posts (Table 1).

### Presentation of the CoEd Technology

**Figure 2:**
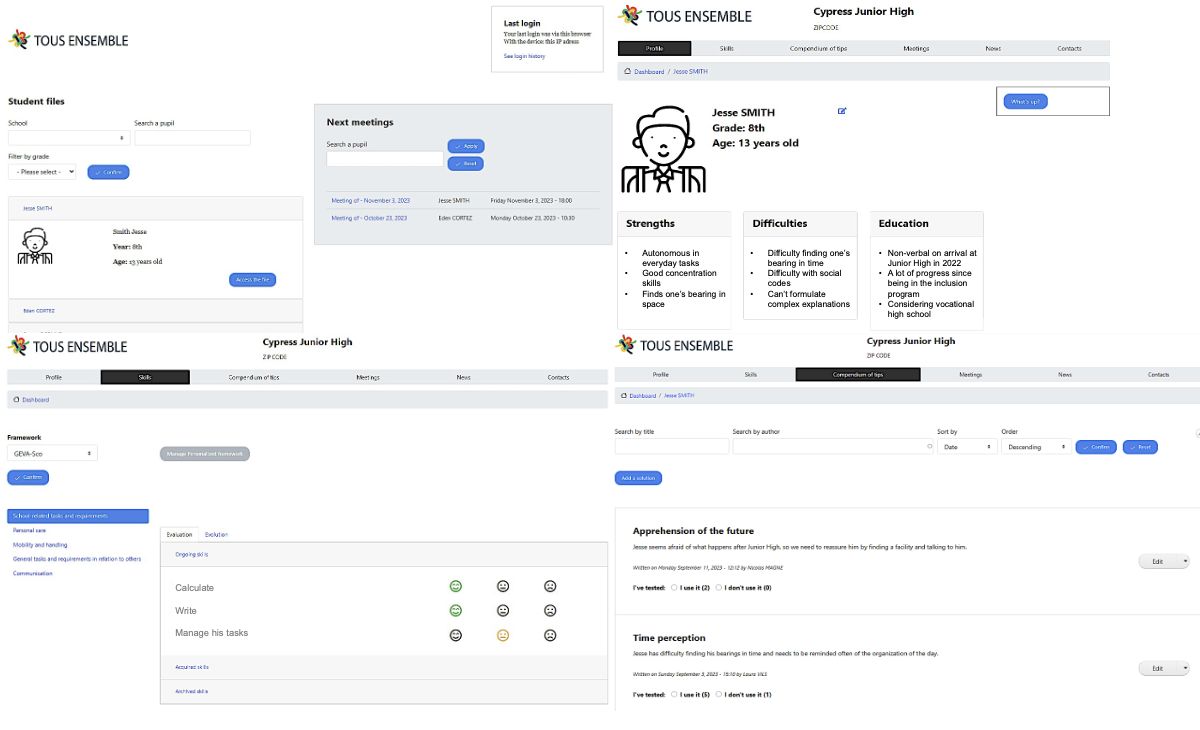
Screenshots of different tabs from the CoEd app.

Specific to the French school context, the CoEd application (Figure 2) is accessible through a web browser and allows users to view and edit one or multiple student files. Users need to be registered by the administrator and can log in with an email and password. Roles—parent, teacher, or external professional—are assigned during registration, offering equal file access rights. A designated moderator role, open to any team member by consensus, is suggested for content moderation.

Once logged in, the user can access the student's file, which consists of 6 tabs: profile, skills, meetings, compendium of tips, news, contacts.

As an identity card, the profile provides key main information to know about the child. A “What’s up?” block allows users to be informed of new content in the folder.

The skills section allows users to review the child’s skill assessment. Two skill referentials are provided: (1) a formal IEP evaluation (ie, Geva-Sco scale for French IEP) and (2) a custom skill referential, where users can determine the categories and skills they wish to assess according to the child’s profile.

The meetings tab allows user awareness of past and upcoming meetings about the child. This section also provides an interface to automatically generate reports about past meetings in PDF format, which are accessible by all users.

On the compendium of tips tab, users can share tips, aids, and adjustments that they successfully used with the child in particular situations. They can receive feedback from the other team members.

The news section is dedicated to punctual and rapid information sharing, such as daily events or feedback and particular events.

On the contacts tab, all team members related to the child’s folder are identified, including their role in the team and contact information.

### Procedure

When the participants were assigned to the CoEd intervention condition, the researchers created the child’s page and the users’ logins on the CoEd application. The participants may provide initial general information to be put on the child’s page by the researchers. At the beginning of the study and after collecting baseline measures, the participants of the CoEd intervention condition received a user manual and video tutorials to help them handle the application, and additional training could be provided by the researchers if needed. Once the study began, the participants were asked to use the application at least once a week but were free to use it as often as they wanted.

The participants from the control group did not receive any intervention and were asked to function as usual.

All questionnaires were administered through online forms. Every participant received an identification number, which was used to anonymize the data collected. At the date of measurement (baseline, 3-month follow-up, 6-month follow-up), participants were instructed by mail to complete the questionnaires. The data collection was monitored by the researchers, and the participants who did not answer were reminded once a week by mail or phone. If participants remained unreachable for more than 6 weeks, they were labeled as “lost” and excluded from the trial.

The data collected through forms and the CoEd application were stored on a secure server at the Inria research center and anonymized using participants’ identification numbers. Only researchers working on the project are authorized to consult the collected data.

### Statistical Analysis

#### Power

We did not find previous studies assessing the impact of a digital device on IEP teams’ relationships and communication in order to perform a power analysis. However, some behavioral interventions with the same target were published and reported moderate effect sizes. Thus, we based our power analysis on an effect size of *d*=0.600.

#### Planned Statistical Analyses

We plan to conduct pre-post mean comparison analysis on primary (RQ1) and secondary outcomes (RQ2 and RQ3) to assess within and between-team effects of the intervention. We will conduct within-team analyses using 1-way ANCOVA to compare pre and postintervention differences separately for each team’s experimental and control groups. This approach helps identify specific changes within teams and assesses differential effects between groups. Additionally, a between-team analysis will treat all participants as a single population, using repeated-measures ANCOVAs or linear regression analyses to examine the overall intervention effect across teams. This global analysis disregards team distinctions to assess the intervention’s overall impact. In addition, using ANCOVA will allow us to control for potential bias coming from the variability in technological skills (Computer Usage Questionnaire; see Table 1) as well as any variable that may compromise the homogeneity across groups. Missing data will result in the exclusion of related observations, and dropouts will be monitored to explore the reasons.

By combining these methods, we aim to provide a comprehensive evaluation of the psychological intervention’s effectiveness, considering both within-team variation and overall effects.

To assess the user experience (RQ4), only the participants of the CoEd intervention condition answered the user experience questionnaire. These data will be analyzed through descriptive statistics and compared with questionnaire-related benchmarks.

## Results

The study was funded in 2021 with public funds from Conseil Régional d’Aquitaine and the Fondation International pour la Recherche Appliquée au Handicap (FIRAH), Klesia, and Comité National Coordination Action Handicap (CCAH).

**Figure 3:**
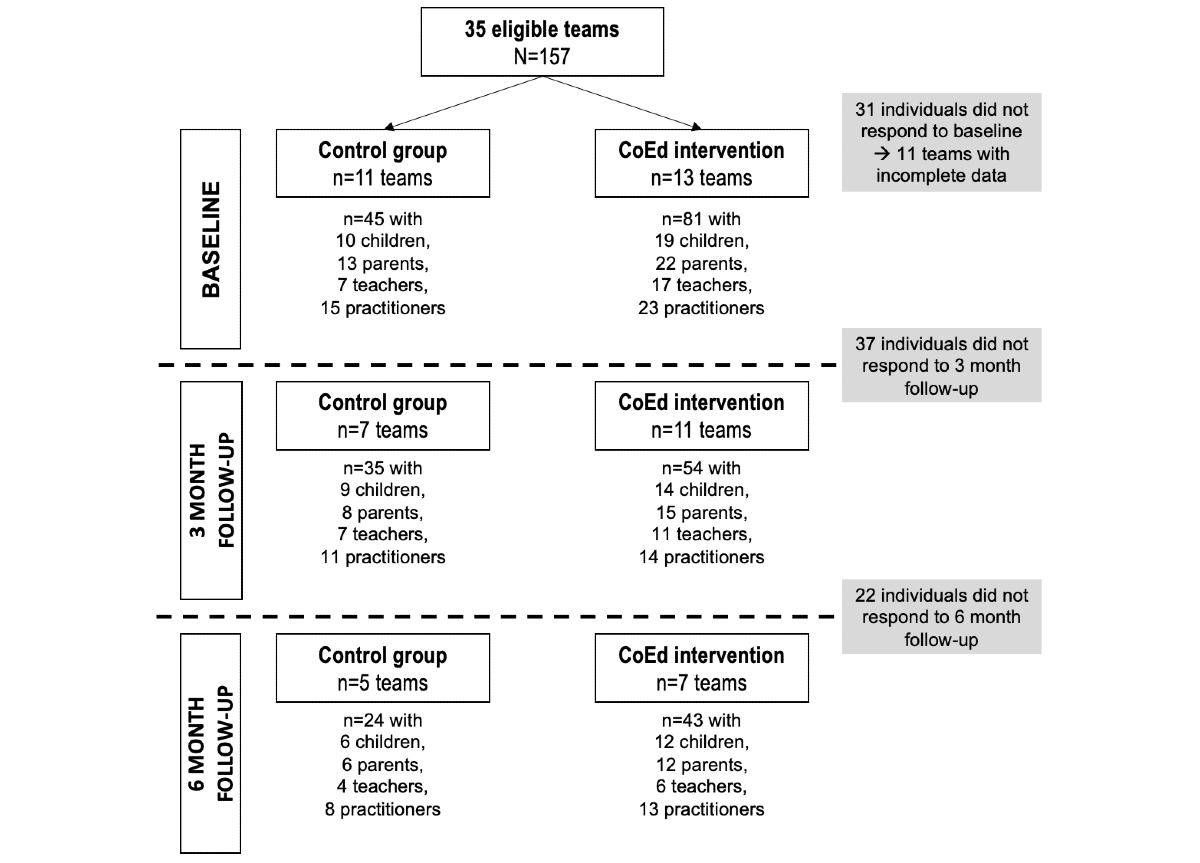
Flowchart of participant recruitment.

Figure 3 shows the flowchart of the recruitment process. We initially recruited 37 eligible teams who were allocated to either the experimental or control group, which resulted in a sample size of 157 participants (2-7 people per team). At baseline, 31 individuals did not complete the questionnaires, leading to the exclusion of 11 teams due to incomplete data. In September 2023, the recruitment ended with 13 teams participating in the experimental condition (n=82) and 11 teams in the control condition (n=45). The experimental group is larger than the control group due to the nature of our action-research approach. Participants and partners expressed a strong preference for being included in the intervention to benefit from the CoEd app, viewing control group assignment negatively. To address this while maintaining rigorous assessment, we included more participants in the experimental group, ensuring sufficient data for analysis and respecting the ethical considerations of action research.

At the end of data collection (June 2024), a total of 59 individuals had dropped out: They stopped responding to the questionnaires and did not reply to our follow-up attempts. Consequently, there were 89 remaining participants at the 3-month follow-up and 67 participants at the 6-month follow-up. Data cleaning and analysis began in March 2025, and results are expected by June 2025. We anticipate that the findings may be ready for publication by the end of 2025, although this timeline remains provisional.

## Discussion

### Expected Results and Comparison With Prior Work

This research protocol was developed to empirically evaluate the impact of a digital technology solution on collaborative relationships among stakeholders in inclusive education. Conducting a field study with a quasi-experimental protocol will provide true empirical support of the technology’s utility and effectiveness for fostering stakeholder collaboration. Through the targeted primary outcomes, we particularly expect an improvement in the quality of interpersonal relationships as well as more congruent cross-stakeholder attitudes toward school inclusion while empowering each stakeholder in their capabilities to achieve their own goals as educator and to support child in their IEP (RQ1). We also expect a beneficial effect of using the CoEd application on child-related outcomes (RQ2) and on the quality of life of stakeholders and children (RQ3).

Meyer et al [35] identified 6 digital projects and a strategy using an existing social network, all at different development stages. Of these projects, 3 used participatory design, 4 conducted needs analyses, and 3 were field-tested. However, none included a control group nor aimed to evaluate the impact on school inclusion, IEPs, stakeholder interactions, or health and well-being dimensions. From this review, it becomes evident that conducting quasi-experimental field studies and involving a substantial number of educational teams and various stakeholders will be an essential contribution. Building on this, our protocol seeks to provide robust evidence for a digital co-education tool designed with user-centered design methods and approaching a gold standard methodology.

### Strengths and Limitations

The CoEd application has already completed all the design and user testing in order to reach the step of empirical evidence thanks to this study (see [36] for the design study). Furthermore, our field study included evaluations that cover more broadly other critical indicators for co-educators, such as their mental health using the burden and quality of life assessments. There is strong evidence of the great influence of perceived self-efficacy on a person’s well-being [59,60]. Hence, we will be able to explore the links between the primary outcomes (interpersonal relationships, attitudes toward school inclusion, and self-efficacy) and some health measures. For the children, the collaboration benefits from technology could promote, by the bounds effect, their sense of school inclusion, school-life satisfaction, and overall well-being through an effect on parent-teacher relationships and collaboration between stakeholders [6,7,61-63]. This technology could also facilitate the implementation of IEPs by leveraging each stakeholder’s expertise [6,8] and, as a result, increasing the educational achievement (academic and social objectives) for each child.

Additionally, the log data from the CoEd application will provide insights on its actual usage across time and the targeted primary outcomes. For instance, RQs could address the traditional dilemma between quality and quantity of exchanged information, or content analyses could even document interpersonal tensions and their potential relation with a degraded partnership in the team and vice versa.

For the intervention group using the CoEd application, we are collecting data on usability, technology acceptance, and usage factors. This assessment is of great importance in studies examining the impacts of technology-based interventions, as usability and effectiveness are intrinsically related, and is part of most studies evaluating this kind of intervention [37].

These secondary indicators address the needs of project stakeholders (inclusive school policymakers, school organizations, teachers, practitioners, parents, and children with SEND) for successful participatory achievements and pave the way for innovative research, such as exploring relationships between CoEd acceptance and effectiveness in inclusive school environments. Most of the participants of this study are people who did not participate in the design steps of the project. Therefore, the bias from “being both designer and evaluator” is limited, and the results from this study will provide insight on the efficacy of using a citizen science approach in the domain of inclusive education. This study combines a traditional top-down approach (ie, expectations relative to specific primary criteria for a prescriptive purpose) with a bottom-up approach driven by the field expertise of the main stakeholders of school inclusion. As a reminder, the study outcomes have been cobuilt thanks to focus group methods involving actual stakeholders as well as researchers in the field (see Multimedia Appendix 2 for details about the focus group sessions). This citizen science approach (albeit risky) contributes to bridging science and practice, facilitating rapid societal impact, and emerging new research issues [64,65]. The results from this study could also contribute to documenting the effectiveness of citizen science and participatory design methods in designing innovative technologies tailored to the specific needs of people, especially in health and disability domains [24,65,66].

From our combined approaches, the study consortium encountered a few issues that reinforced the study-related challenges, especially with children with SEND and with a technology-based intervention. First, major challenges were related to the quasi-experimental study design in which the CoEd intervention condition is compared with a control condition where there are fewer teams. We paid great attention to matching the 2 groups with respect to the factors known for influencing the primary outcomes as well as possible. These factors include the size of the team, demographic factors, and the SEND of the child. Nonetheless, we are aware that such a matching method does not neutralize possible biases related to differences in the group size. We plan to explore the impact of any variable that is not matched between groups to investigate its impact on the intervention’s effect. A second study challenge was the great variability in the participants’ technological skills in the CoEd condition. Indeed, computer skills and prior experience with technology will necessarily influence the experience with using a digital tool and, in turn, modulate the intervention’s effects. We set up a standardized training phase for the use of CoEd application according to training use scenarios and provided both written and video tutorials to help people handle the CoEd application. In addition, the questionnaire on technological skills will be used as a covariable to explore the impact of this interindividual variability on the intervention’s effects. Another study challenge was associated with the increasing digitalization of schools, whether in teaching, student support, or parental involvement. Since the COVID-19 pandemic, this digitalization has significantly increased, especially in the field of education [67]. However, the extensive digitalization during the COVID-19 pandemic brought issues related to digital anxiety and data protection [68], increased screen time, and the effects on well-being and interpersonal relationships [69-71]. In this project, these themes were central during design and development steps, as it involved processing data concerning children with NDD. This point was addressed during the participatory design steps and proved to be a very important one for future users [36]. During the development steps, we made data security a priority, so that users can use the system with complete confidence (compliance with regulations, security certification). Finally, the last challenge our research had to confront was related to the context of digital development in France. The French government has introduced a new digital tool to bridge the gap between decision-making bodies for educational obligations and resources allocated for the implementation of an IEP and schools. This deployment has led teachers to increase digital data input on academic difficulties and IEP accommodations. However, this deployment has caused confusion with CoEd and could lead some teachers to limit their participation, as some data would need to be entered both in CoEd and the governmental digital tool. However, there are important differences between the CoEd application and the governmental tool: The latter is more oriented toward archiving aims and digitalization of the IEP, while the CoEd application promotes free sharing of experiential knowledge from each stakeholder, which indirectly contributes to IEP implementation and follow-up. In addition, the CoEd application allows stakeholders to communicate with each other about daily events, which is not covered by the governmental tool. In this context, the CoEd application can be viewed as an aid for following IEP objectives and to better support the use of the governmental app through complementary features.

Despite all these challenges, the outcomes of the CoEd study will yield significant insights on the benefits of a digital collaborative tool for teaching students with SEND. It will also yield some insights on the communication factors influencing the health of stakeholders and children. The study will also inform the feasibility of technology-based personalization of IEP and will likely teach some useful lessons for future technology-based field studies regarding empowering stakeholders to teach children with SEND.

### Dissemination Plan

Results from our study will be disseminated via scientific publications and conferences. In addition, as we previously included decision-makers of the French public policy of inclusive schooling in the CoEd design phases, we made sure that the solution is ethically, technologically, and financially sustainable in the public services. This will provide a solid foundation for a business model in case there is a startup to market the technology.

A second perspective is to explore the feasibility of disseminating the CoEd application outside of the French frontiers, by scaling up in other countries. However, there are cultural and structural differences between countries, particularly regarding inclusive education. This international dissemination will necessitate an additional needs analysis and adaptations to the application for the specific context of other countries [72]. A first step will be to explore the feasibility of disseminating the application in French-speaking countries with a similar cultural background (eg, Belgium), thereafter moving to wider internationalization with other European countries. This internationalization strategy is certainly ambitious and will necessitate a new needs analysis and adaptations of the CoEd application.

### Conclusion and Future Directions

Although further research is needed, the findings of the CoEd project will provide the first real evidence-based study in the field of educational technology for social environments supporting children with SEND with their IEPs.

To move the robustness of the evidence forward, it will be necessary to consider conducting a trial using strict randomized controlled trial methods. In the same vein, the question of a large-scale intervention exists. As also mentioned, our ecosystemic and participatory approach has led us to work directly with the key stakeholders in inclusive education in order to both design an upstream CoEd application and evaluate it downstream. Consequently, a scaling up in other countries for the evaluation of the CoEd application will be required for better proof of the interest for an interactive web application for a co-education process.

## Appendix

Multimedia Appendix 1 SPIRIT checklist for this study.

Multimedia Appendix 2 Description of Focus group for selecting study outcomes.

## Abbreviations

ASD: autism spectrum disorder
CCAH: Comité National Coordination Action Handicap
COERLE: Comité Opérationnel d'Évaluation des Risques Légaux et Éthiques
FIRAH: Fondation Internationale pour la Recherche Appliquée au Handicap
GDPR: General Data Protection Regulation
IEP: individual education plan
Inria: French National Institute of Informatics and Mathematics
NDD: neurodevelopmental disorders
RQ: research question
SEND: special educational needs or disabilities
SPIRIT: Standard Protocol Items: Recommendations for Interventional Trials

## Data Availability

Upon study completion, the data sets generated during or analyzed during this study will be available from the corresponding author on reasonable request.
