# Supplementary material for: Evaluating a Web-Based Application to Facilitate Family-School-Health Care Collaboration for Children With Neurodevelopmental Disorders in Inclusive Settings: Protocol for a Nonrandomized Trial
Source: JMIR Res Protoc. 2025 Apr 17;14:e63378. doi: 10.2196/63378 (PMC12046275; doi:10.2196/63378)
Supplement: Multimedia Appendix 2 [file resprot_v14i1e63378_app2.docx]

# Appendix 2 – Report on Focus group session to co-decide outcomes of the field study

## Method

Two focus group sessions were organized in June 2021, with parents, teachers and health care professionals. As it was at the end of the Covid-19 pandemy, the two sessions were conducted through visioconference and using Wooclap to animate the sessions and collect the particpants’ answers (open and close questions).

Each session began with researcher and participant introducing themselves, and a presentation of the CoEd app (features, examples of use). Then, participants were invited to discuss potential success and failure indicators, which are of the most important for them if the CoEd app was deployed on the field. These discussions were animated and aided by the researcher, who follow a focus group guide (as a semi-structured interview).

There was two open-ended questions : 1. “for you, what would be the success indicators for this app ?” and 2. “for you, what would be the failure indicators for this app?”. On these questions, participants were invited to answer on Wooclap, and then their answer were classed following emerging themes.

Then, a close-ended question was asked : “how would you rank the proposed indicators in terms of priority?”, with a list of indicators proposed by researcher with regards to the literature.

## Results

### Open-ended question 1 : Success indicators

| Theme | Participants’ answers |
| --- | --- |
| At school | Increased amount of inclusion time |
|  | Child’s communication skills |
|  | Increased amount of stakeholders’ communication |
|  | Better knowledge about the child |
|  | Improved child’s well-being in their school |
|  | Better support during transitions |
| At home | Better awareness on child’s daytime |
|  | More information on school works |
|  | Reduced behavioral decompensation |
|  | Enjoying of school |
| Family-School relationship | More sharing of tools |
|  | More positive meeting time |
|  | Reduced number of errors due to lack of information |
|  | More information relay |
|  | Fewer sources of tension |
| Relationship with health care professionals | More sharing of tools |
|  | Better alignment of practices |
|  | Better awareness of the child’s particularities |

### Open-ended question 2 : Failure indicators

| Theme | Participants’ answers |
| --- | --- |
| Parent-Teacher conflicts | Not using the app (lack of time or willing) |
|  | Too much information |
|  | Lack of communication |
|  | Fear of not being legitimate |
|  | Fear of judgement |
| Child’s well-being | Not bringing the child to their full potential |
|  | Exchanges of criticism through the child |
| Inter-professional relationships | Refusal of sharing practices |
|  | Lack of communication |
|  | Fear of not being legitimate |
| Care and support | Lack of confidentiality |
|  | Less care and support |

### Close-ended question : Suggested indicators ranking among researchers’ selection

| Ranking | Indicator |
| --- | --- |
| 1 | Child’s well-being |
| 2 | Parent-Teacher Relationships |
| 3 | Feeling of Self-efficacy |
| 4 | School-related child’s well-being |
| 5 | Attitudes towards inclusive education |
| 6 | Congruency of perception among stakeholders |
| 7 | Perceived stress |
| 8 | Perceived burden |
